# Refractive results with the use of AT.Lisa intraocular lens (2008-2015)

**Published:** 2016

**Authors:** Filip Mircea, Nicolae Miruna, Filip Andrei, Antonescu Cristina, Dragne Carmen, Triantafyllidis Grigorios, Moisescu Raluca, Lutic Irina, Ungureanu Ileana, Teodorov Anamaria

**Affiliations:** * AmaOptimex, Eye Clinic, Bucharest, Romania

**Keywords:** IOL, multifocal, toric, emmetropia, AT, Lisa

## Abstract

The purpose of the study was to evaluate the refractive results on a large cohort of patients who were implanted spherical or toric multifocal IOL’s for cataract surgery or for refractive purpose. Preoperative refractive investigations included auto refractometer topography, pentacam, contact and noncontact biometry and many non-refractive investigations. The target in multifocal IOL usage was emmetropia and it was achieved in most cases. Ametropia occurrence involved correction in different ways.

AMA Optimex Clinic receives educational support from Carl Zeiss Instruments SRL. There are no other financial interests.

## Statistics

Between August 2008 and August 2015, 1168 AT.Lisa IOLs were implanted in our clinic. Now, 21% of the total implants are AT.Lisa (over 200 per year) and the number is increasing. Also, in October 2012, toric AT.Lisa IOLs started being used as well. In this paper, we studied and presented the refractive result on a smaller but representative number of patients.

**Fig. 1:**
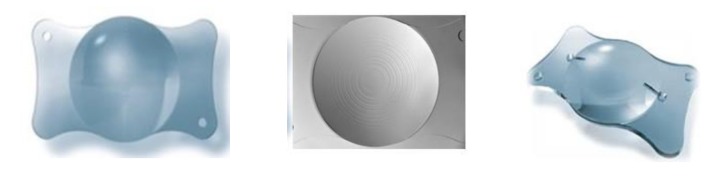
AT. Lisa IOLs

## Purpose

The purpose of this paper was to evaluate the refractive results, on a representative cohort of 102 cases of spherical or toric multifocal IOLs used in cataract surgery or for refractive cases. Evaluating the patient’s satisfaction as well as reaching the target goal of emmetropia were also tried to be accomplished.

## Our Study

Our study was conducted on 102 cases operated by 3 surgeons in our clinic, from June 2014 until June 2015. There were 46 (45,09%) females and 56 (54,91%) males, with the age varying from 27 to 77 years old, operated for cataract (46-77 years old) and for RLE (27-60 years old, also PreLEX cases).

We included in our study 60 cataracts (58,82%) and 42 RLE (41,18%) were also included in the study and 32 AT.Lisa bifocal IOLs (31,37%) and 54 AT.Lisa trifocal IOLs (68,83%) were implanted. In addition, there were 16 AT.Lisa toric IOLs: 2 bifocal (13,72%) and 14 trifocal (86,28%). 66,66% of the cataract patients were operated on both eyes, 95,24% being from the RLE patients.

## Preoperative assessment

When the decision to operate was taken, the patients underwent a complete ocular examination including biomicroscopy, refraction keratometry, intraocular pressure, Schirmer test, corneal topography, Pentacam, pachymetry, Humphrey perimetry, macular OCT, ocular ultrasound and biometry with both IOL Master and Ultrascan. The keratometry was performed with the refractometer, topographer and IOL Master [**1**,**2**].

The patients were advised to stop the use of contact lenses for a week before the investigations. The formulas used to calculate Acri.LISA power during biometry were in short eyes (<22mm) Haigis, Holladay II, Hoffer Q; in normal eye length (22-25mm) SRK-T, Haigis, Holladay II, Hoffer Q; in long eyes (>25mm) Haigis, Holladay II [**1**].

## Patient’s selection criteria

The patients who benefited most from the implantation of a AT.Lisa IOL were those with a positive attitude and with an active life, patients interested in new things and with great motivations, patients with high standards, but definitely not perfectionists, cooperative patients who could also make compromises; also patients who suffered greatly from the psychological stress of presbyopia [**3**].

The dominant eye was operated first. There is an easy test to find the dominant eye: when asked to fix his eyes on his thumb, the patient will always use the dominant eye. The recommendation is for bilateral implantation for best clinical outcomes.

## Exclusion criteria

The patients who were not suitable for the implantation of a multifocal IOL were hypercritical patients, patients with unrealistic expectations, patients with monofocal IOL in fellow eyes (optional), patients with ocular and general diseases, patients with amblyopia or uncorrectable astigmatism [**4**].

## Contraindications for AT.Lisa implantation

From the wide range of general diseases, a contraindication for an AT.Lisa IOL implant is the existence of a severe Diabetes Mellitus, as retinal detachment is expected and contrast sensitivity can be severely affected due to maculopathy.

Mental retardation is also an impediment as mental adaptation is required.

Ocular pathology such as severe diabetic retinopathy, corneal diseases (opacification, scarring, keratoconus, severe sicca syndrome), retinal dystrophies, severe vitreous opacities, strabismus,uveitis,amblyopia,retinitispigmentosa are not to be associated with the implantation of a multifocal IOL such as AT.Lisa [**4**].

## The surgery

The surgery and the implantation of an AT.Lisa IOL are practically the same as in any other cataract/ RLE procedure. Eye infections prophylaxis is done preoperative with topical and systemic antibiotics. The anesthesia is usually retroocular, but can also be topical for RLE in high myopias. The capsulorhexis should be small or normal, oval shaped with the longer axis vertically for a better implantation, especially of the proximal part of the IOL. In the unfortunate case of a capsular tear, a sulcus implantation is to be desired. The implantation of the AT.Lisa IOL was performed with a Bluemix 180 injector [**2**,**3**].

The fellow eye should be operated as soon as possible for a better neural adaptation with the IOL: next day or next week.

## Postoperatively and complications

The topical treatment was prescribed for 14 days using tropicamide, antibiotic and anti- inflammatory drops. Sometimes the tropicamide was tapered after one week to one drop per day at nighttime.

We recommended intense eye activity for both intermediate and near vision!

In the case of PCO, Yag should be performed after the first month.

If biometrical errors occurred, an action was taken after 6-12 months with EXCIMER; the patient was informed about this possibility before surgery.

Another important complication is Macular Cystoid Edema, which is treated in a classical way [**1**,**3**].

## Patients’ satisfaction

In order to evaluate the patients’ satisfaction after surgery, a little questionnaire inquiring about the quality of the far, intermediate and near vision, the existence of haloes and glare, was used. Patients were asked to make an overall estimation in the end.

The far vision was perceived as good in 86,27% of the cases, satisfactory in 13,72% and none of the patients was unhappy (0%). In the case of the intermediate vision, 86,27% of the patients estimated it as good, 7,85% as satisfactory, none was unhappy (0%); 5,88% of the patients (4 cases) did not use a computer, so, they could not appreciate it quite well. The near vision was good in 80,39% of the cases, satisfactory in 19,60% of the cases and no patient was unhappy (0%). The night vision was evaluated as good for 61,76% of the patients, satisfactory for 29,41% (haloes) and 8,82% were unhappy.

## Results and conclusions

It was estimated that with the use of AT.Lisa IOLs, the patient’s satisfaction was generally reached, spectacle independence and good night vision being achieved.

In the overall estimation, 90,19% of the patients were happy, in 7,84% of cases, the result was satisfactory and 1,96% were unhappy due to miodesopsias.

It is important to know that there has to be a psychological support for the patient before and after surgery and a good communication regarding any aspect, including the surgery and the adaptation required afterwards. A technically perfect surgery does not mean that the patient will be satisfied with the result because all the side effects slowly disappear in the months after surgery. Even if the side effects do not disappear completely, they do not disturb the patients after a while. We do not emphasize on spectacle independence, but on current activities and YES, sometimes glasses may be needed [**5**]. 
